# Individual competence predominates over host nutritional status in Arabidopsis root exudate-mediated bacterial enrichment in a combination of four *Burkholderiaceae* species

**DOI:** 10.1186/s12866-022-02633-8

**Published:** 2022-09-17

**Authors:** Javier Ignacio Cillero, Pablo Andrés Henríquez, Thomas Warwick Ledger, Gonzalo Andrés Ruz, Bernardo González

**Affiliations:** 1 Laboratorio de Bioingeniería, Facultad de Ingeniería y Ciencias, Universidad Adolfo Ibáñez, Diagonal Las Torres, 2700 Santiago, Chile; 2 Center of Applied Ecology and Sustainability (CAPES), Santiago, Chile; 3 Facultad de Economía y Empresa, Universidad Diego Portales, Santiago, Chile

**Keywords:** Arabidopsis, Bacterial growth, *Burkholderiaceae*, Co-culture, Root exudates

## Abstract

**Background:**

Rhizosphere microorganisms play a crucial role in plant health and development. Plant root exudates (PRE) are a complex mixture of organic molecules and provide nutritional and signaling information to rhizosphere microorganisms. *Burkholderiaceae* species are non-abundant in the rhizosphere but exhibit a wide range of plant-growth-promoting and plant-health-protection effects. Most of these plant-associated microorganisms have been studied in isolation under laboratory conditions, whereas in nature, they interact in competition or cooperation with each other. To improve our understanding of the factors driving growth dynamics of low-abundant bacterial species in the rhizosphere, we hypothesized that the growth and survival of four *Burkholderiaceae* strains (*Paraburkholderia phytofirmans* PsJN, *Cupriavidus metallidurans* CH34, *C. pinatubonensis* JMP134 and *C. taiwanensis* LMG19424) in *Arabidopsis thaliana* PRE is affected by the presence of each other.

**Results:**

Differential growth abilities of each strain were found depending on plant age and whether PRE was obtained after growth on N limitation conditions. The best-adapted strain to grow in PRE was *P. phytofirmans* PsJN, with *C. pinatubonensis* JMP134 growing better than the other two *Cupriavidus* strains. Individual strain behavior changed when they succeeded in combinations. Clustering analysis showed that the 4-member co-culture grouped with one of the best-adapted strains, either *P. phytofirmans* PsJN or *C. pinatubonensis* JMP134, depending on the PRE used. Sequential transference experiments showed that the behavior of the 4-member co-culture relies on the type of PRE provided for growth.

**Conclusions:**

The results suggest that individual strain behavior changed when they grew in combinations of two, three, or four members, and those changes are determined first by the inherent characteristics of each strain and secondly by the environment.

**Supplementary Information:**

The online version contains supplementary material available at 10.1186/s12866-022-02633-8.

## Background

Plants are sessile organisms adapted to respond to environmental changes in many ways. One of the main spaces of adaptation and dynamics response is the rhizosphere, defined as the volume of soil under the influence of plant roots [1], where many phenomena, such as water and nutrient absorption and modulation of root microbiome, occur [2]. The plant’s secretion of Plant Root Exudates (PRE) is important in the highly dynamic interrelationships between plant roots and soil microorganisms [3–6]*.* PRE are complex solutions of low molecular weight, organic compounds, and macromolecules secreted by the plant roots that play a significant role in the rhizospheric environment. PRE components modulate the rhizospheric microbial communities by providing macro (i.e., carbon and nitrogen sources), micronutrients, and chemical signals ranging from antibiotic to quorum-sensing molecules [2, 7, 8].

The rhizosphere microbial communities are composed of different taxa [6, 9] that interact among them and with plants [10–12], establishing beneficial, neutral, or pathogenic effects [4, 5, 9]. The study of the structure, composition, and dynamics of rhizosphere microbial communities has been a field of great interest during the last decade, showing that these communities are highly dependent on plant species, soil type, ecotype, and potential stress conditions, among other variables [5, 6, 9, 11, 12]. For example, an exhaustive comparative 16S rDNA metadata study that the rhizosphere showed a predominance of copiotroph bacteria regardless of origin or environment that responds more rapidly to changes in nutrient inputs than the bulk soil-related communities. Although progress in describing the global dynamics of these communities has advanced and new techniques have been developed to study the complex dynamics of the rhizosphere; there are still questions and challenges in describing the behavior of these communities, especially when we delve into the dynamics of phylogenetically close members, with potentially similar metabolic niches, and how it is affected by their interactions [10–12].

To study how PRE affects individual and communities’ rhizobacteria behavior, the usual strategy is to collect exudates from soil or hydroponic plant cultures, which is the most reproducible and straightforward way to study its effects on nutrient deficiencies and stress factors [7, 8]. This strategy has been used in various plants, from crops to grasses. Among them is *Arabidopsis thaliana,* which has been widely studied to characterize the rhizosphere microbial community’s assembly and recruitment and how these Arabidopsis*-*associated communities change with plant development, plant genotypes, and biotic/abiotic stress [5, 11–13].

Studies based on Next Generation Sequencing technologies have defined the *A. thaliana* core microbiome as composed of members of a few phyla, i.e., *Pseudomonadota*, *Actinomycetota*, *Bacillota,* and *Bacteroidota*, found both in the rhizosphere and phyllosphere [11–13]. Among *Pseudomonadota*, alpha, beta, and gamma classes are predominant, but the distribution of low-rank taxa is quite uneven within these classes. For example, *Paraburkholderia*, *Cupriavidus,* and *Ralstonia* genera belonging to the beta proteobacterial *Burkholderiaceae* family exhibit low to very low abundance levels in *A. thaliana* microbiomes [11–13].

However, species belonging to *Paraburkholderia* and *Cupriavidus* genera interact with plants in several ways. For example, *C. pinatubonensis* JMP134 degrades a variety of aromatic compounds [14], several of them components of PRE [7, 8], with these catabolic abilities playing a role in plant colonization and plant protection [15]. *C. metallidurans* CH34, with tolerance to a wide range of metals [16], can promote growth and protect Arabidopsis from copper effects [17], and *C. taiwanensis* LMG19424 fixes nitrogen in nodulating plants [18]. In turn, *P. phytofirmans* PsJN is a well-known plant growth-promoting bacterium able to establish rhizospheric and endophytic colonization on several plants, including Arabidopsis [19–22]. Thus, despite their low abundance, *Burkholderiaceae* can have a significant impact on *A. thaliana* growth, which may also be influenced by variations in population numbers within specific compartments, as suggested by the correlation of a lower rhizosphere colonization ability for quorum sensing mutants of strain PsJN and a reduction in the growth promotion effects on the plant [23].

It should be noted that most of the plant-microbe interactions have been studied under laboratory conditions with isolated strains. In contrast, in nature, microorganisms interact negatively (competition, predation, parasitism) or positively (synergism, mutualism, commensalism) with each other within dynamic microbial communities [24], which adapt and evolve depending on the conditions in which the plants thrive [25]. In addition, little has been studied on the interactions between low-abundant rhizospheric organisms as the ones described above. Even knowing that some of these organisms play an essential role in the functioning and health of the plant [26]. The knowledge of these low-abundance members’ behavior and their interactions with the plant or with each other remains scarce. To understand the factors driving interactions on this low abundance rhizospheric bacteria, we conducted a study to assess how the four *Burkholderiaceae* strains indicated above survive and grow on *A. thaliana* root exudates. This PRE were obtained from Arabidopsis plants grown at different plant age and nitrogen availability to address potential variability in the collected exudates. These conditions were selected as proxies for developmental stages (young versus mature states) and nutrient availability (N is the main plant growth limiting nutrient). The results showed that the behavior of these *Burkholderiaceae* strains depended more on the presence or absence of other strains (microbial interactions) than the PRE used for growth.

## Results

### The individual growth of *Burkholderiaceae* strains on Arabidopsis exudates

**Fig. 1:**
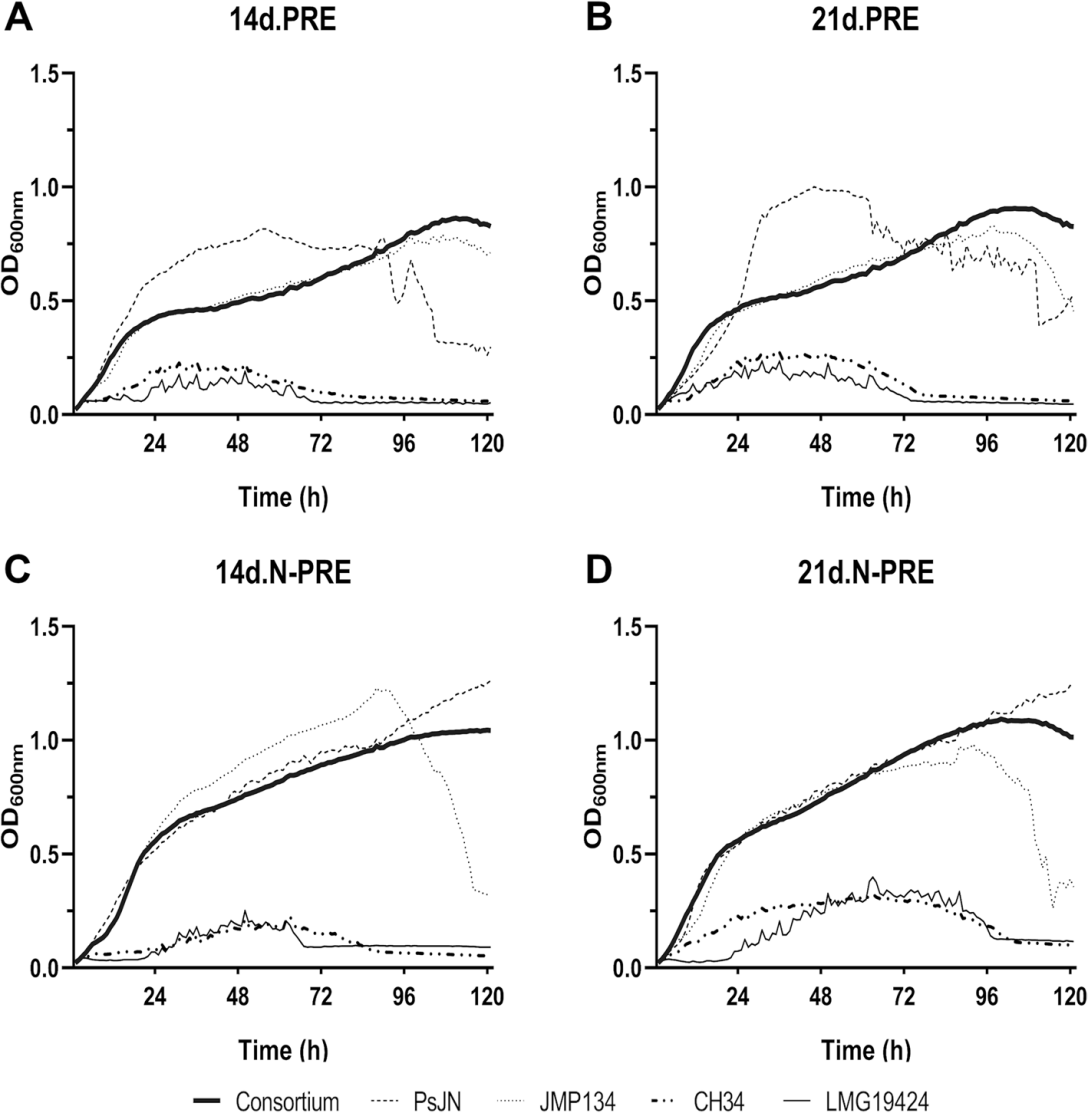
Growth curves of the four *Burkholderiaceae* strains on *Arabidopsis thaliana* root exudates. Growth curves were obtained after 120 h in culture tests carried out with *A. thaliana* root exudates (PRE) collected on day 14, or 21, with plants grown on standard (PRE) or N-limiting (N-PRE) conditions: (**A**) 14d.PRE; (**B**) 21d.PRE; (**C**) 14d.N-PRE, and (**D**) 21d.N-PRE. Each growth curve corresponds to the average of three replicates of cultures inoculated with each individual strain alone, or the 4-member combination of *Paraburkholderia phytofirmans* PsJN, *Cupriavidus pinatubonensis* JMP134, *C. metallidurans* CH34, and *C. taiwanensis* LMG19424. Standard deviations were lower than 5–10% and are not shown for clarity

The ability of these four strains to use each of the four PRE as the sole carbon and energy source was first tested. To rule out a possible interference due to the use of sucrose during the germination of *Arabidopsis*, residual levels of sucrose were measured for each PRE (14d.PRE, 21d.PRE, 14d.N-PRE, and 21d.N-PRE) with average values of 0.078 ± 0.022; 0.076 ± 0.029; 0.022 ± 0.004; and 0.071 ± 0.050% p/v respectively (Table 1, first row), which represent values that showed no difference with this PRE. In agreement, tests performed in liquid cultures containing 0.1% p/v sucrose showed no growth for the four strains. Growth tests on *A. thaliana* root exudates showed that *P*. *phytofirmans* PsJN and *C. pinatubonensis* JMP134 reached statistically higher population levels than *C. metallidurans* CH34 and *C. taiwanensis* LMG19424, with each of the four PRE (Fig. 1). On average, *P*. *phytofirmans* PsJN and *C. pinatubonensis* JMP134 grew 5.4 times faster than strains CH34 and LMG19424 on N limited exudates, whereas *P*. *phytofirmans.*

**Table 1:** Gross composition of exudates

	14d.PRE	21d.PRE	14d.N-PRE	21d.N-PRE
Residual Sucrose (%p/v)	0.078 ± 0.022	0.076 ± 0.029	0.022 ± 0.004 *	0.071 ± 0.050
Phenolics (μg/mL)	100.24 ± 17.24	105.95 ± 23.0	100.64 ± 5.1	107.38 ± 14.87
Carbohydrates (mM)	2.89 ± 0.83*	1.10 ± 0.56	1.13 ± 0.43	1.15 ± 0.23
Proteins (μg/mL)	0.37 ± 0.0001	0.37 ± 0.0001	0.36 ± 0.0002	0.37 ± 0.0002
Total Organic Carbon (TOC) (mg/L)	54.26 ± 2.56	54.60 ± 2.57	56.27 ± 4.65	28.90 ± 6.59*

Five different organic compounds were measured to explore possible differences between each plant root exudate. For more information on the techniques and protocols used in each case, refer to the Methods section. Each row shows the average of three technical replicas with their respective standard deviation. Comparisons between each exudate were made using Student’s t-test, and the significant differences are indicated with asterisks (*).

PsJN and *C. pinatubonensis* JMP134 grew 4.3 and 1.4 times faster than *C. metallidurans* CH34 and *C. taiwanensis* LMG19424 in 14d.PRE and 21d.PRE, respectively, excepting that *C. metallidurans* CH34 proliferated 1.4 times faster than *C. taiwanensis* LMG19424 in 21d.-PRE. This comparison showed that all strains started the stationary phase after 24–30 h, except for *C. metallidurans* CH34 growing on 14d.N-PRE, and *C. taiwanensis* LMG19424 growing on 14d.N-PRE and 21d.N-PRE, where stationary phases were only achieved later than 48 h of culture (Fig. 1). Stationary phases lasted more than 72 h for *P*. *phytofirmans* PsJN and *C. pinatubonensis* JMP134, or around 48 h for *C. metallidurans* CH34 and *C. taiwanensis* LMG19424. Maximum growth yields were higher on N-PRE than in PRE. Death phases were observed for *P*. *phytofirmans* PsJN in 14d.PRE and 21d.PRE, and *C. pinatubonensis* JMP134 in 14d.N-PRE and 21d.N-PRE, whereas *C. metallidurans* CH34 and *C. taiwanensis* LMG19424 showed a slower decline in bacterial cell numbers after 72 h in all conditions (Fig. 1).

The gross composition of these PRE was determined and is shown in Table 1. Five measurements were performed to determine residual sucrose levels, total phenolic, total carbohydrates, total protein, and total organic content. The measurements show no significant differences between the PRE on residual sucrose levels, total phenolic, and protein content. On the other hand, the total carbohydrate content found was significantly higher (double) in 14d.N-PRE from the other three PRE. Finally, total organic carbon contents were substantially lower (half) on 21d.PRE from the other three PRE.

### Better together? Growth of combinations of *Burkholderiaceae* strains in Arabidopsis root exudates

Potential cooperation or competition interactions among bacteria were determined in growth cultures inoculated with a mixture of the four strains, starting (T0) at the same concentration (0.1 OD_600nm_). When these strains were grown together, the same growth pattern was observed with all tested PRE, with maximum yields ranging from 1.0–1.2 OD_600nm_ and generation times of 7.1 h and 7.9 h on 14d.PRE and 21d.PRE, respectively, and of 9 h and 6.3 h on 14d.N-PRE and 21d.N-PRE, respectively (Fig. 1). The shapes of the co-culture growth curves were essentially like those observed for the individual growth curves of *P*. *phytofirmans* PsJN and *C. pinatubonensis* JMP134 in all PRE, except for the higher or lower growth levels transiently observed with *P*. *phytofirmans* PsJN grown on standard conditions PRE, and the death phases of *C. pinatubonensis* JMP134 occurring with N-PRE (Fig. 1). Bacterial abundances were determined for each strain growing in the 4-member combination at final growth times (120 h) (Table 2, Generation 1). Bacterial abundances of *C. pinatubonensis* JMP134 were 1–2 orders of magnitude higher than those of *P*. *phytofirmans* PsJN and *C. taiwanensis* LMG19424, except for the latter in 21d.NPRE. In contrast, absolute abundances of *C. metallidurans* CH34 were 1–3 orders of magnitude lower than the other three strains in all PRE. These results suggest that, at the end of the co-culture, *C. pinatubonensis* JMP134 was the main responsible for growth performance within the 4-member co-culture. This hypothesis was further studied using the k-means clustering algorithm, expecting *C. pinatubonensis* JMP134 to cluster with the 4-member co-cultured growth.

**Table 2:** Final abundances of the *Burkholderiaceae* strains growing as a 4-member combination on *Arabidopsis thaliana* root exudates, after six sequential transfers

	Generation	Final abundance (Log_10_CFU/mL)			
		PsJN	JMP134	CH34	LMG19424
14d.PRE	1	8.13 ± 0.20	8.90 ± 0.24	8.10 ± 0.27	8.13 ± 0.20
	2	4.95 ± 0.27	5.41 ± 0.35	4.93 ± 0.30	4.95 ± 0.21
	3	4.87 ± 0.22	4.77 ± 0.19	0.00 ± 0.00	4.60 ± 0.0
	4	0.00 ± 0.00	0.00 ± 0.00	0.00 ± 0.00	0.00 ± 0.00
	5	0.00 ± 0.00	0.00 ± 0.00	0.00 ± 0.00	0.00 ± 0.00
	6	0.00 ± 0.00	0.00 ± 0.00	0.00 ± 0.00	0.00 ± 0.00
	7	0.00 ± 0.00	0.00 ± 0.00	0.00 ± 0.00	0.00 ± 0.00
21d.PRE	1	10.81 ± 0.20	12.01 ± 0.15	9.03 ± 0.09	10.81 ± 0.20
	2	9.63 ± 0.29	10.7 ± 0.13	8.05 ± 0.22	9.63 ± 0.22
	3	8.76 ± 0.11	8.29 ± 0.23	7.06 ± 0.35	7.52 ± 0.15
	4	7.52 ± 0.18	8.82 ± 0.20	5.85 ± 0.05	7.45 ± 0.22
	5	4.69 ± 0.21	4.81 ± 0.21	0.00 ± 0.00	3.70 ± 0.29
	6	7.72 ± 0.26	8.60 ± 0.60	0.00 ± 0.00	0.00 ± 0.00
	7	6.57 ± 0.20	7.45 ± 0.29	0.00 ± 0.00	0.00 ± 0.00
14d.N-PRE	1	9.48 ± 0.34	11.85 ± 0.20	8.92 ± 1.16	9.48 ± 0.34
	2	9.08 ± 0.72	10.59 ± 0.35	8.33 ± 0.35	7.74 ± 0.07
	3	7.30 ± 0.32	7.27 ± 0.31	6.80 ± 0.31	7.27 ± 0.23
	4	7.21 ± 0.33	7.37 ± 0.34	6.50 ± 0.14	6.78 ± 0.22
	5	7.43 ± 0.35	7.25 ± 0.52	6.25 ± 0.38	7.06 ± 0.40
	6	6.71 ± 0.33	7.27 ± 0.26	5.61 ± 0.25	6.74 ± 0.26
	7	7.33 ± 0.20	7.35 ± 0.33	5.67 ± 0.42	7.32 ± 0.47
21d.N-PRE	1	10.85 ± 0.24	12.12 ± 0.18	8.83 ± 0.23	11.87 ± 0.29
	2	6.61 ± 0.29	6.67 ± 0.22	5.51 ± 0.26	6.58 ± 0.11
	3	7.55 ± 0.26	7.61 ± 0.26	6.30 ± 0.27	7.52 ± 0.27
	4	8.71 ± 0.28	8.65 ± 0.41	5.77 ± 0.12	9.41 ± 0.61
	5	7.14 ± 0.21	6.99 ± 0.22	5.36 ± 0.17	6.62 ± 0.15
	6	6.59 ± 0.17	6.53 ± 0.11	4.87 ± 0.13	6.47 ± 0.18
	7	5.55 ± 0.20	6.23 ± 0.14	3.76 ± 0.22	5.27 ± 0.33

Final abundances determined by selective plate culture of *Paraburkholderia phytofirmans* PsJN, *Cupriavidus pinatubonensis* JMP134, *C. metallidurans* CH34, and *C. taiwanensis* LMG19424 after growth on *A. thaliana* root exudates (PRE) collected at day 14, or 21, with plants exposed to standard or N-limiting conditions: 14d.PRE, 21d.PRE, 14d.N-PRE, and 21d.N-PRE. Each value represents averages, and standard deviations from three replicates

**Fig. 2:**
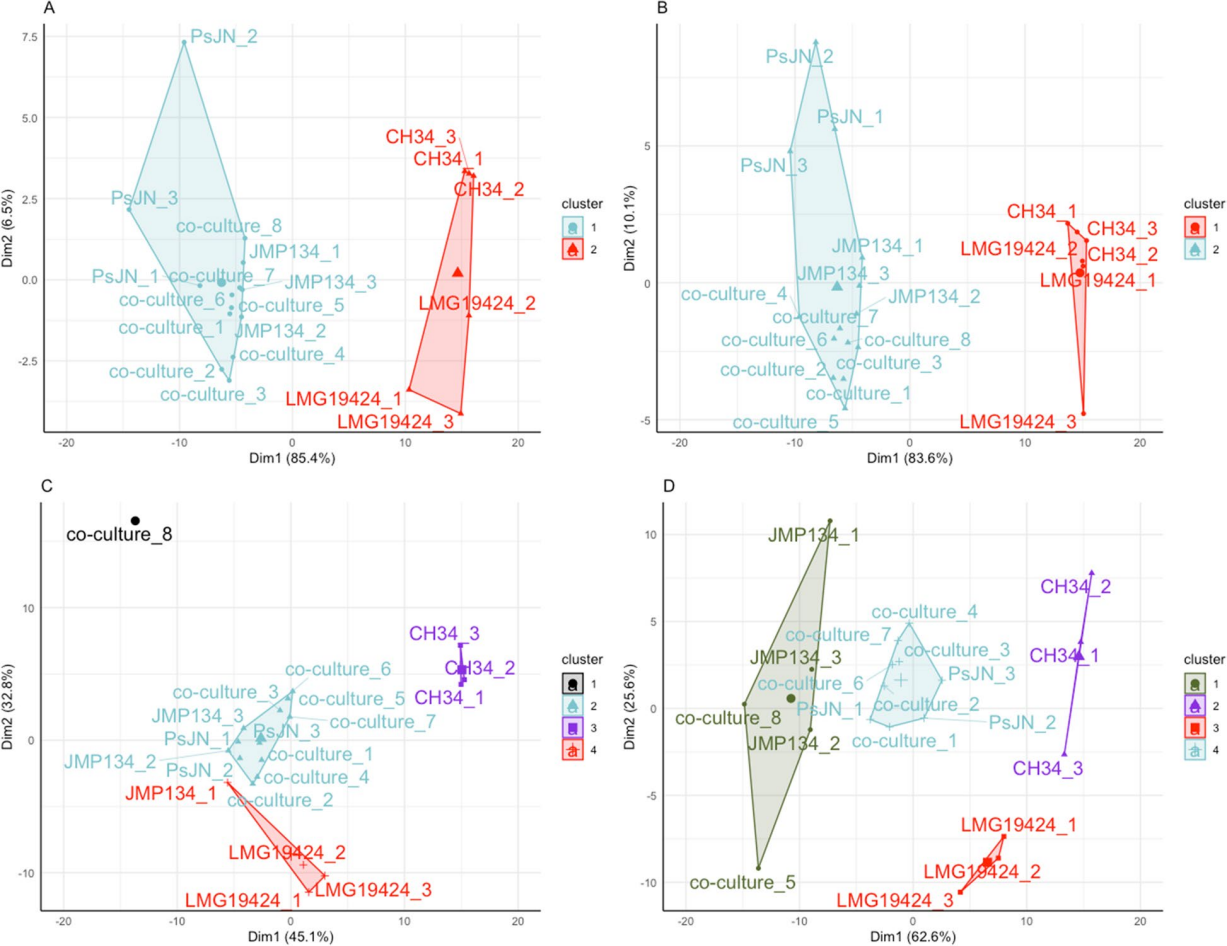
First generation cluster discrimination. Graphic representation of cluster determination through K-means. Each panel (**A**) 14d.PRE; (**B**) 21d.PRE; (**C**) 14d.N-PRE, and (**D**) 21d.N-PRE, represent the output of the K-means clustering algorithm. On each panel, 20 growth curves were analyzed to obtain the clusters: eight replicates of the initial 4-member combination (Consortium_1 to Consortium_8) and three replicates for each individual culture: PsJN_1 to PsJN_3 for *Paraburkholderia phytofirmans* PsJN; JMP134_1 to JMP134_3 for *Cupriavidus pinatubonensis* JMP134; CH34_1 to CH34_3 for *C. metallidurans* CH34, and LMG19424_1 to LMG19424_3 for *C. taiwanensis* LMG19424. Clusters that contain the 4-member combination are colored light blue. A larger symbol is presented in each cluster centroid

The growth pattern found for the 4-member co-culture and their individual growths were compared to analyze if the growth dynamics of the 4-member co-culture resembled that of any individual bacteria and, therefore, some of them dominate over the others in the co-culture (Fig. 2). It was observed that the co-culture grouped with *P*. *phytofirmans* PsJN and *C. pinatubonensis* JMP134 in all PRE (Fig. 2A-C), except 21d.N-PRE, where *C. pinatubonensis* JMP134 grouped only with two co-cultures replicates (co-culture_8 and co-culture_5), and *P*. *phytofirmans* PsJN grouped with the remaining six replicates (Fig. 2D). On the other hand, significant changes among PRE were explored. The results show that only two clusters were determined for 14d.PRE and 21d.PRE (Fig. 2A&B), the first composed of *P*. *phytofirmans* PsJN, *C. pinatubonensis* JMP134, and the co-culture, and the second formed by *C. metallidurans* CH34 and *C. taiwanensis* LMG19424. On the other hand, the cluster determined for PREs obtained from plants under N-limiting conditions was less homogeneous. On the one hand, in 14d.N-PRE (co-culture_8 was considered as an outsider [Fig. 2C]), three clusters were determined, while 21d.N-PRE displayed four significant clusters (Fig. 2D). These results demonstrated a clear effect of the type of exudates with both individual strains and co-culture. Also, they corroborate the previous observation that growth curves in the co-culture were mainly influenced by *P*. *phytofirmans* PsJN and *C. pinatubonensis* JMP134.

It is worth mentioning that no positive or negative effects between pair combinations grown under two standard laboratory conditions were found. LB and R2A plate cross strike tests revealed no growth inhibition halos. In addition, growth and survival tests performed in spent media (i.e., liquid culture media after growth of one of these four strains) in LB and 5 mM succinate Dorn minimal medium showed no decrease/increase in survival (measured as CFU/mL) or growth (OD_600nm_) after 48 h of incubation. These results indicate that no inhibitory compounds nor growth-enhancing molecules were produced upon the development of the first strain.

### Exploring microbial interactions through combinatorial co-culturing

**Fig. 3:**
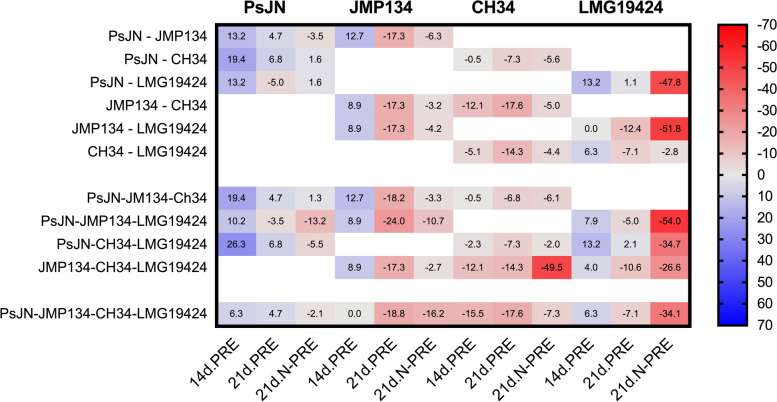
Percentage of variation in viable cell numbers of *Burkholderiaceae* strains growing on Arabidopsis root exudates when single cultures are compared with co-cultures combinations. Percentages of variation in viable cells were calculated after comparison of single cultures values with those for pairs, trios, and quartet combinations of *Paraburkholderia phytofirmans* PsJN, *Cupriavidus pinatubonensis* JMP134, *C. metallidurans* CH34, and *C. taiwanensis* LMG19424 cultures on *A. thaliana* root exudates (PRE) collected at day 14, or 21, with plants exposed to standard or N-limiting conditions (14d.PRE, 21d.PRE and 21d.N-PRE). The color code bar corresponds to decreases (red) or increases (blue)

To further explore interactions between these strains that would explain the final abundances of the 4-member co-culture (Table 2, Generation 1) and the different aggrupation found in the cluster analysis (Fig. 2), combinatorial co-culture growth tests were carried out to determine viable cell counts. Since 14d.N-PRE and 21d.N-PRE absolute bacterial abundances were similar (Table 2, Generation 1), co-culturing tests were carried out only with 14d.PRE, 21d.PRE and 21d.N-PRE. Individual growth levels were compared with those determined in pairs, trios, and the 4-member combinations (Additional File 1), and the corresponding percent variations in viable cell numbers were calculated (Fig. 3). PRE heavily modified cell numbers of each strain growing in combinations. Decreases in the abundances were more frequent than increases (Fig. 3), indicating that inhibitory interactions predominated. Percent variation increases were observed for *P*. *phytofirmans* PsJN (6 to 26%) co-cultures grown on 14d.PRE, and to a lesser degree for *C. pinatubonensis* JMP134 (7–9%) and *C. taiwanensis* LMG19424 (4–13%), with no essential differences if the co-culture consisted of pairs, trios, or the full quartet, except in two cases (*C. taiwanensis* LMG19424 when is paired with *C. pinatubonensis* JMP134, and JMP134 on the quartet arrangement) where *C. taiwanensis* LMG19424 was part of the co-culture (Fig. 3). Although *C. metallidurans* CH34 had decreased growth on any combination (1–15%), this effect was more significant on co-cultures with strains LMG19424 and JMP134. The 21d.PRE negatively affected the growth of *C. pinatubonensis* JMP134 (14–24%), *C. metallidurans* CH34 (7–18%), and *C. taiwanensis* LMG19424 (7–12%), except for minor increases (1–2%) for *P*. *phytofirmans* PsJN, but not in the presence of *C. pinatubonensis* JMP134, which may be related to the better performance of *P*. *phytofirmans* PsJN in this exudate. In contrast with 21d.PRE, the 21d.N-PRE, consistently decreased growth for all strains when tested in co-cultures (Fig. 3), with *C. taiwanensis* LMG19424 being the most affected (3–52%), *C. metallidurans* CH34 and *C. pinatubonensis* JMP134 decreasing between 3 and 10%, and *P*. *phytofirmans* PsJN showed decreases (2–13%), or slight increases (1–2%).

### Sequential transfer dynamics of four-member co-culture

**Fig. 4:**
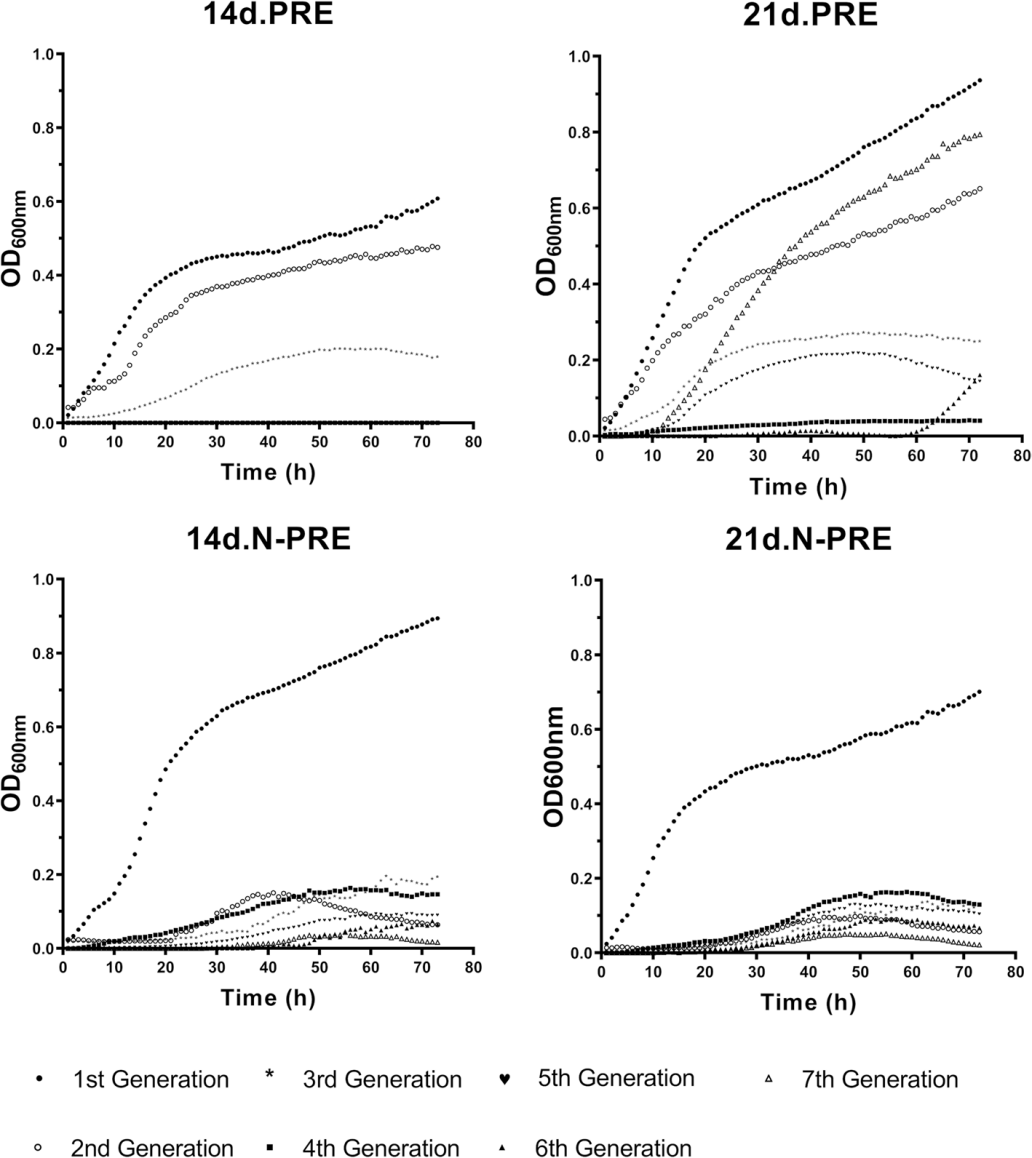
Growth curves of the 4-members *Burkholderiaceae* co-culture on *Arabidopsis thaliana* root exudates for seven generations: Growth curves obtained after 72 h in culture tests carried out with *A. thaliana* root exudates (PRE) collected at day 14, or 21, with plants grown on standard or N-limiting conditions: (**A**) 14d.PRE; (**B**) 21d.PRE; (**C**) 14d.N-PRE, and (**D**) 21d.N-PRE. Each growth curve corresponds to the average of eight replicates of the 4-member co-culture of *Paraburkholderia phytofirmans* PsJN, *Cupriavidus pinatubonensis* JMP134, *C. metallidurans* CH34, and *C. taiwanensis* LMG19424. Standard deviations were lower than 5 or 10% and are not shown for clarity

To study potential fitness changes in time, the abundances of these *Burkholderiaceae* strains were determined after six sequential transfers, i.e., seven generations (Fig. 4). Decreases in maximal growth were observed after the first sequential transfer (Fig. 4A, C&D), except for 21d.PRE after the 4th generation, where an increase in growth was detected (Fig. 4B). Also, the shapes of the growth curves changed with longer lag phases: 12.5-, 15.8-, 19.2-, and 10-fold average increases for 14d.PRE, 21d.PRE, 14d.N-PRE, and 21d.NPRE, respectively, and slower generation times: 1.2-, 2.7-, 3.0-, and 1.5-fold average increases for 14d.PRE, 21d.PRE, 14d.N-PRE and 21d.N-PRE, respectively. There were, however, some differences between 14d.PRE, 14d.N-PRE, and 21d.N-PRE. For the former, changes were steadily observed through the initial generations, e.g., maximal 14d.PRE growth yields of 0.61, 0.48, 0.2, and 0.15 OD_600nm_, were detected (Fig. 4A), whereas for the latter, a sharp decrease was detected soon after the first transfer, e.g., maximal 14d.N-PRE growth yields of 0.9, 0.15, 0.19, 0.16, 0.07, and 0.21 OD_600nm_, were observed (Fig. 4C). For 14d.PRE and 14d.N-PRE from the 4th generation onward, the 4-member co-culture never recovered, and no growth could be detected (Fig. 4A&C). On the other hand, with 21d.PRE after the 4th generation, the 4-member co-culture has a non-stable behavior (Fig. 4B). The 5th generation showed a 12 h lag phase followed by a log phase with a generation time of 3.53 h, reaching a maximum OD_600nm_ value of 0.22. The 6th generation showed a long lag phase (56 h) followed by a short log phase that reached a maximum OD_600nm_ of 0.16 at 72 h. Finally, the 7th generation showed a 10 h lag phase followed by a log phase of 8.4 h and a maximum OD_600nm_ of 0.8 at 72 h (Fig. 4B). On the other hand, the 4th member co-culture showed growth in all generations in N-PRE, regardless of the age of the plant from which the exudate was collected (Fig. 4 C&D). These results showed that growth on N-PRE decreased steadily over time (e.g., maximal growth on 21d.N-PRE from 4th generation: 0.08, 0.07, 0.04, 0.02).

To analyze if the abundances of the members of this co-culture changed over time, viable cell counts were determined at the end of each culture (Table 2). Results for 14d.PRE showed complete loss of viable cells since the 4th generation, except for *C. metallidurans* CH34 which disappeared in the 3rd generation. Individual abundances were determined after growth on 21d.PRE showing that the four strains decreased in viable cell numbers after sequential transfers. *P*. *phytofirmans* PsJN and *C. pinatubonensis* JMP134 remained at significant levels even after the sixth transfer, with *C. pinatubonensis* JMP134 always showing higher abundance levels (Table 2). In contrast, *C. metallidurans* CH34 and *C. taiwanensis* LMG19424 completely disappeared after the 5th and 6th generation, respectively. A different pattern was observed with both N-PRE. The four strains remained viable at significant levels even after the sixth transfer, although *C. metallidurans* CH34 exhibited levels two orders of magnitude lower than the other strains, with *C. pinatubonensis* JMP134 showing at least one order of magnitude higher levels than the other strains. However, viable cell counts diminished after sequential transfers with the four strains, especially in 21d.N-PRE. Taken together, these results indicate that the inability of this 4-member combination to sustain growth on PRE depends on the type of PRE and the bacterial interactions.

## Discussion

The differences observed in growth on PRE for the four species can be explained because PRE produces the proliferation or decrease of certain microorganisms in the rhizospheric environment due, among other effects, to a co-adaptation process [27, 28], resulting from a dynamic and complex environment where better-adapted microorganisms could grow [5, 28]. Results recently published by dos Santos et al [29] confirm this idea. They reported that not only stimulation of certain metabolic pathways is observed in *Gluconacetobacter diazotrophicus* when is co-cultivated with Arabidopsis, but also that Arabidopsis constitutively exudates compounds that facilitate the plant-bacteria interaction. Environmental conditions in which plants develop produce different exudate compositions [8, 30]. Both the plant’s age and nutrient availability generate exudation changes, which are reflected in different microbial behavior [31]. This study showed that nutrient availability causes greater differences (better yields and longer log phases) than plant age. Although the exudate changes the growth pattern, the inherent microbial capacities for individual competence were prevalent, and strains like *P*. *phytofirmans* PsJN or *C. pinatubonensis* JMP134 better behave on any PRE than *C. metallidurans* CH34 and *C. taiwanensis* LMG19424. Together with the above, the results showed that the oldest plant exudates do not present a decrease in growth parameters to any of the bacteria, suggesting that the nutrient consumption carried out by the plant during this time frame does not generate a reduction in growth, but rather that the exudation pattern is different.

Gross composition comparisons among the four PRE revealed no significant differences with a couple of exceptions. The values reported here generally agree with similar determinations in other plants [3, 32, 33]. Therefore, the effects of PRE on bacterial growth described here may probably arise from differences in a narrower subset of organic compounds.

The individual behavior within a co-cultured group of bacteria has been studied mainly to enrich the area of consortium engineering for creating synthetic consortia. Understanding each bacterium’s collective behavior allows finding insights into how synthetic co-cultures could be engineered to create technological applications [34, 35]. In the present study, it should be considered that interactions (both positive and negative) among bacteria in co-cultures can be produced directly (bacterium-bacterium) or indirectly (mediated by PRE components and their bacterial metabolism). The latter possibility seems more probable as growth curves in synthetic media (Additional File 3) showed no effects. The 4-member co-culture studied here showed similar behavior to *C. pinatubonensis* JMP134 on PRE and to *P*. *phytofirmans* PsJN on N-PRE, which was corroborated by clustering analysis. Also, these analyses showed that the behavior of *C. metallidurans* CH34 and *C. taiwanensis* LMG19424 on N-PRE were different enough to cluster separately, demonstrating again that the 4-member co-culture and the individual growth were affected by the PRE origin. In this context, both the environment (i.e., PRE) and each bacterium’s inherent capacities favor that *C. pinatubonensis* JMP134 and *P*. *phytofirmans* PsJN dominate over the other *Cupriavidus.* This kind of information would allow for modulation of the environment so that, for example, would be possible to design a dynamic co-culture of *P*. *phytofirmans* PsJN and *C. pinatubonensis* JMP134 where the first one dominates the culture over *C. pinatubonensis* JMP134 in 21 days old plants. However, co-culture abundance analysis showed that the abundance distribution between the different PRE varies, which agrees with previous reports [2, 35]. The fact that *C. pinatubonensis* JMP134 was always the most abundant member and *C. metallidurans* CH34 was always the least abundant, indicates the inherent characteristics of each bacterium that makes them better or worse adapted to the co-culture condition and highlights the importance of considering the co-culture as a dynamic system were both, the environment, and the bacterial characteristics, influence the behavior of the co-culture.

One of the difficulties involved when studying the dynamics and composition of a co-culture arrangement of bacteria is that the ecological relationships within their members scale linearly each time a strain is added to a co-culture [35]. The combinatorial analysis performed here showed that some bacteria benefited (high final bacterial abundance) from being in co-culture in any combination (*P*. *phytofirmans* PsJN and *C. pinatubonensis* JMP134 at 21d.PRE), while others had their abundance diminished (low final abundance) by the presence of any additional strain on the culture. It is important to mention that predatory or toxic behaviors (no positive effects in growth) among these strains were not evidenced in any combination studied on standard solid cultures or spent liquid cultures. Therefore, at least for standard rich or minimal media negative/positive interactions such as, for example, those mediated by volatile organic compounds, antibiotics, or other bioactive compounds, are not observed among these four strains, at least in non-PRE culture media.

These observations illustrate that the dynamics of environmental co-cultures are determined by the ecologic relationships between co-cultured members and therefore, these complex dynamics should be considered when a novel or natural consortium is intended to consolidate. Although the low abundance of the members does not always correlate with a diminished metabolic activity or influence over the consortium, it has been shown that rare elements in natural consortia offer redundancy elements, control of overpopulation by key niche occupation, among other capabilities [26], and therefore, the growth dynamics exposed here are important to consider and further study when a microbial consortium is intended to be used on biotechnological or agronomics applications.

The observed differences between the 4-members co-culture growth parameters on N-PRE versus PRE suggest an important influence on exudation composition when plants grew under nutrient deficient conditions. Similar results were reported in rice growth patterns under different levels of nitrogen availability. The researchers demonstrated that adequate levels of the macronutrient generate greater carbon availability in soil organic matter, attributable to greater carbon rhizodeposition [36]. However, it has been shown that the addition of nitrogen sources to the soil has no effects on the bacterial abundance, or bacterial community composition compared with unfertilized soils. Therefore, it has been hypothesized that the system is influenced by direct or indirect plant effects on the soil [1]*.* As an example, Kavamura et al. [37] reported that predicted functional pathways in wheat rhizobacteria under no nitrogen fertilization, have higher terpenoid-related metabolism markers and lower amino acid markers. These could be related to our results given the varied metabolic capacities of these *Burkholderiales* strains especially to degrade aromatic compounds [14, 15], which are medium abundance PRE components [2, 4, 7, 8]*.* In contrast, sugar metabolism is quite uncommon in these strains [30] which suggests a differential use of PRE components compared with other rhizosphere members.

An interesting phenomenon observed in the sequential transfer was that co-cultures grew less and with a different dynamic immediately after the first transfer. One possibility to explain such a decrease is that the first generation exhibited higher growth because the first inoculum was made with cells grown separately and then co-cultured. Another option is related to the time the selection was conducted. It has been shown that selection times highly influence microbiome selection experiments [38, 39]. It should also be noted that from the second generation onward, lag phase duration increases supporting the idea that the growth stage in which the transfer takes place influences how the next generation will behave.

## Conclusions

A differential ability to grow on PRE was found for each *Burkholderiaceae* species. The best-adapted strain to grow in PRE was *P*. *phytofirmans* PsJN, with *C. pinatubonensis* JMP134 growing better than the other two *Cupriavidus* strains. Individual strain behavior changed when they succeeded in combinations of two, three, or four members. The plant age when root exudates were collected and whether exudates were obtained after growth under N limitation conditions have some effect on bacterial performance. The type of PRE affected the outcome of the sequential transfer of the 4-member co-culture. Bacterium – bacterium direct interactions can be essentially disregarded.

## Methods

### Experimental design

**Fig. 5:**
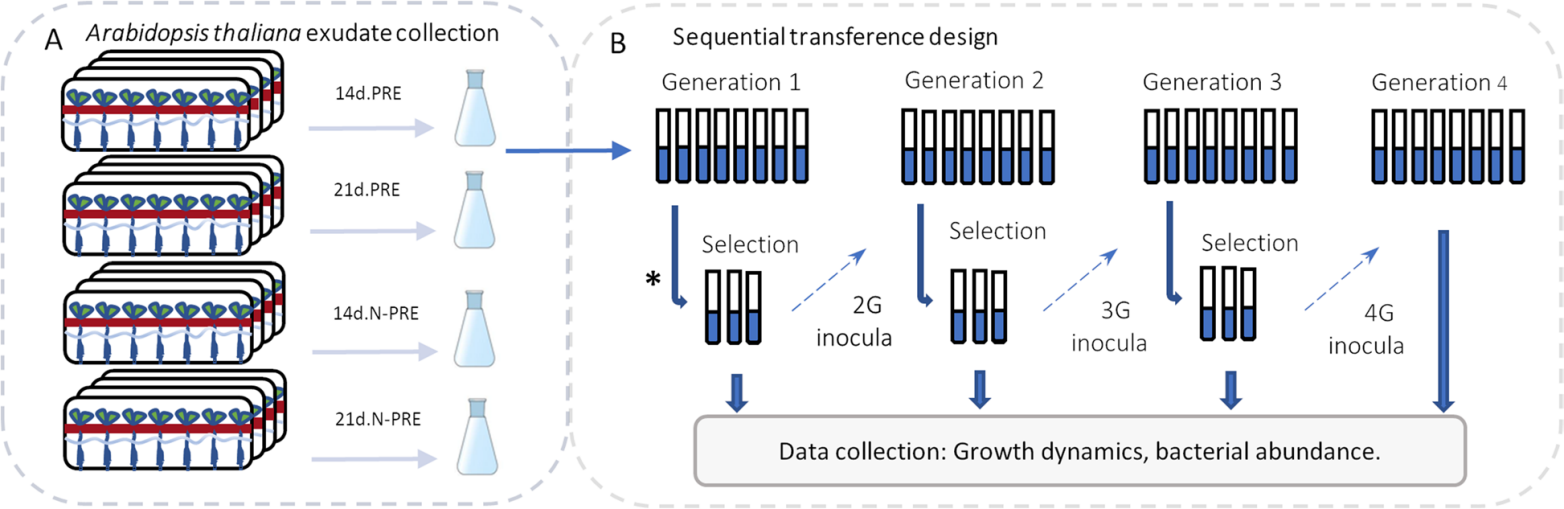
Experimental design. Procedure to obtain *Arabidopsis thaliana* root exudate pools (**A**), used in the sequential transference experiment (**B**). Plant root exudates were collected from plants growing under standard or nitrogen limitation conditions after 14 or 21 days; 14d.PRE and 21d.PRE, and 14d.N-PRE and 21d.N-PRE, respectively. Eight replicates of the individual or combined inocula of *Paraburkholderia phytofirmans* PsJN, *Cupriavidus pinatubonensis* JMP134, *C. metallidurans* CH34, and *C. taiwanensis* LMG19424 were grown for 120, or 72 h. Growth curves, viable cell counting, and bacterial abundances were then determined. Three of the eight replicates were selected to inoculate the next generation. This process was repeated until the seventh generation. For more details on the selection procedure, see Additional File 2

The fitness of these four strains in the rhizosphere environment was explored, testing the ability to use PRE as a sole carbon and energy source. The effect of potential PRE variability was tested using PRE collected at two plant ages and plants grown at standard or limiting N levels. Cooperation or competition among strains was studied in mixed cultures, and fitness stability was addressed by sequential transfers to a new PRE culture media from the same stock. A hydroponic culture system was implemented to produce a gnotobiotic *A. thaliana* PRE batch. A summary of the process is shown in Fig. 5A. Each sterile plastic tray (Phytatrays™, Sigma Aldrich™, Milwaukee, WI, USA) contained a grid where 60 *A. thaliana’s* seeds were sown. This generated a closed system where all plant exudate components are released into the liquid medium. Plants were grown for 14 or 21 days under standard or nitrogen limitation conditions (14d.PRE, 21d.PRE, 14d.N-PRE, 21d.N-PRE). *Burkholderiaceae* strains were inoculated individually and collectively on each PRE at an equal T_0_ concentration of 0.1 OD_600nm_ regardless of its origin (initial culture, sequential transfer) and then cultured on a microplate incubator spectrophotometer to determine growth levels. Then, a sequential transference experiment was carried out (Fig. 5B). Three of the eight replicates were selected based on those with higher abundances in the 4-member co-culture and with individual growth levels within the median error bars (Additional File 2). The selected replicates were mixed and diluted to 0.1 OD_600nm_ to create the next generation inoculum and then transferred to a fresh pool of the respective PRE. The sequential transfer was performed six times. Generations 1 to 4 were grown for 120 h to have a broad time frame, but later generations (5 to 7) were produced only for 72 h, as after that time, culture stability was observed.

### Bacterial inoculants

*P. phytofirmans* PsJN was obtained from our laboratory stock, whereas *C. metallidurans* CH34, *C. pinatubonensis* JMP134 and *C. taiwanensis* LMG19424 were obtained from the Deutsche Sammlung von Mikroorganismen and Zellkulturen GmbH, Braunschweig, Germany. All strains were grown overnight on Dorn mineral base salt medium [40]*,* with 15 mM succinate to produce the primary cultures for the microplate growth experiment. Other choices as a growth medium to obtain each inoculum, such as Dorn mineral medium plus 10 benzoic acid, Murashige and Skoog (MS) [41] plus 15 mM succinate, or Luria Bertani (LB), were discarded because they gave different growth yields (Additional File 3). The MS medium provides nutrients, micronutrients, and some vitamins [41] and is frequently used for plant growth. Two hundred μL of the corresponding PRE were individually poured into a 96-well microplate well, and cells were inoculated at a final optical density (OD_600nm_) of 0.1 as the initial concentration. All strains were grown for 120 or 72 h on a microplate spectrophotometer (Eon™ Microplate Spectrophotometer, BioTek Instrument, Inc., Vermont, USA), at 30 °C, with a 5 min shaking every hour before the reading. All the strains were inoculated in co-cultures at the same initial OD (OD_600nm_ 0.1). Growth data were obtained and analyzed with Gen5™ v2.0 software (BioTek Instrument, Inc., Vermont, USA). Growth controls were performed in liquid cultures with LB medium, and 100% MS medium (Sigma Aldrich™, Milwaukee, WI, USA), with and without 15 mM succinate. Growth tests in LB and R2A agar plates were routinely performed to detect contamination in finalized experiments. Only rarely do these tests show the presence of unexpected bacterial colonies, and, when they happened, such experiments are discarded.

### *A. thaliana* growth conditions and PRE collection

The study complied with local and national regulations for using plants. *A. thaliana* Col-0 seeds were obtained from the Arabidopsis Biological Resource Center (Ohio State University, Columbus, OH, USA). Sixty stratified and sterilized *A. thaliana* Col-0 seeds were placed on a previously sterilized plastic grid. The grid was then positioned on the gnotobiotic tray supports (Phytatrays™, Sigma Aldrich™, Milwaukee, WI, USA), leaving a gap space that can hold 80 mL of plant growing medium. One hundred percent MS medium (Sigma Aldrich™, Milwaukee, WI, USA), supplemented with (3% p/v) sucrose, was used as a standard condition, and 100% MS modified medium, lacking NH_4_NO_3_ but supplemented with 15 mM of KNO_3_ and (3% p/v) sucrose was used as N limiting condition. The replacement of the N source seeks to help control the added, determining N, using only one N source [42]. In addition, it should be indicated that no replacement of growth medium by fresh 100% MS modified medium was performed. Therefore, the equivalent to 25% N input available in a normal MS medium was the only source of N provided in the N-limiting condition. After 14 or 21 days of culture at 21 °C with 16/8 day/light cycles, plants were removed, and the liquid was collected. Nine replicates were run for each condition, and the exudates were pooled the get required volume. The resulting PRE was filtered (filter unit of 0.22 μm) and stored at 4 °C (Fig. 5A). Sterility was checked in Luria-Bertani and R2A agar plates before and after storage.

### Chemical analysis of collected plant root exudates

Five chemical analyses were performed for each of the four mixed collected exudates. First, a sucrose colorimetric assay was performed with the Sucrose Colorimetric/Fluorometric Assay Kit from Sigma Aldrich™ (Milwaukee, WI, USA) to measure residual levels of sucrose on each PRE. Second, total phenolic content was measured through the Fast Blue BB method [43]. Third, total carbohydrate levels were measured through a phenol-sulfuric acid method [44], which has already been used to measure carbohydrates on plant exudates [17]. Exudate pool samples were diluted at 1:1000 to fit the calibration curves. Forth, total protein quantification was determined by Bradford assay [45] using bovine serum albumin as a protein standard. Fifth, the Chemical Oxygen Demand procedure was performed to indirectly estimate the carbon available on each PRE, following the “Standard Method for examination of Waste and Wastewaters 5220C protocol [46]. All these measurements were performed using three replicates from each exudate pool. A comparison of values was performed by ANOVA.

### Bacterial abundance measurements

The colony-forming units (CFU) measurements were performed using the “drop plate” method for counting viable cells [47]*.* For each strain, a selective medium was designed: *P*. *phytofirmans* PsJN, Dorn basal salt medium (2%) agar plates supplemented with indole-3-acetic acid (10 mM), as a sole carbon and energy source [21]; *C. pinatubonensis* JMP134, Dorn basal salt medium (2%) agar plates supplemented with 2,4-dichlorophenoxyacetic acid (2.5 mM), as a sole carbon and energy source [15]*; C. metallidurans* CH34, R2A (Difco™, Becton-Dickinson & Company, Sparks, Maryland, USA) medium (2%) agar plates with kanamycin (10 μg/mL); *C. taiwanensis* LMG19424, Dorn basal salt medium (2%) agar plates supplemented with 3-hydroxyphenylacetic acid (10 mM), as a sole carbon and energy source, plus gentamicin (10 μg/mL). Selectivity was confirmed for each specific medium as colony growth was only observed in the corresponding culture medium. Due to the inherent differences between these growth media, all CFU measurements were performed 24 h after inoculation to allow complete growth.

### Statistical analysis and K-means algorithm

All the comparisons were appropriately compared using statistical approaches. To simplify the results delivery, such as providing the text of Results and sharper figures, we provide statistical markers only on critical experiments. For more profound information please refer to the raw data supplementary files provided with this manuscript. In summary, some comparisons and statistical analyses are presented below. To compare between each Plant Root Exudate, a one-way ANOVA followed by a Tukey test was performed. To compare the average growth time of each bacterium on the different PRE, we grouped the data and perform a Student t-test to compare. All the statistical analyses were performed using Microsoft Excel software.

A K-means algorithm was used to perform the clustering analysis on the bacterial growth curves. To create the data set, all the points on the growth curves for eight replicates of the 4-member co-culture and three replicates from each individual bacterium culture were selected for each PRE. Briefly, the algorithm works in the following way,

1. Select K points randomly as the initial centroids.
2. Repeat
3. Form K clusters by assigning all points to the closest centroid.
4. Re-compute the centroid of each cluster.
5. Until the centroids do not change

The elbow method was used to determine the numbers of clusters K (Additional File 4).

## Supplementary Information

**Additional file 1. **Viable cell numbers of *Burkholderiaceae* strains from single cultures and co-culture combinations on *Arabidopsis thaliana* root exudates.

**Additional file 2.** Selection of replicates of the 4-member combination to set the next generation inocula.

**Additional file 3 **Growth curves for individual and 4-member coculture of *Burkholderiaceae* strains.

**Additional file 4.** Graphic representation of the elbow method to discriminate number of clusters.

**Additional file 5.**

## Data Availability

The datasets generated and/or analyzed during this study are included as raw data files in Supplementary Information files.
