# Supplementary material for: Individual competence predominates over host nutritional status in Arabidopsis root exudate-mediated bacterial enrichment in a combination of four Burkholderiaceae species
Source: BMC Microbiol. 2022 Sep 17;22:218. doi: 10.1186/s12866-022-02633-8 (PMC9482264; doi:10.1186/s12866-022-02633-8)
Supplement: Supplementary file 1 — Additional file 1. Viable cell numbers of Burkholderiaceae strains from single cultures and co-culture combinations on Arabidopsis thaliana root exudates. [file 12866_2022_2633_MOESM1_ESM.docx]

**Additional File 1. Viable cell numbers of *Burkholderiaceae* strains from single cultures and co-culture combinations on *Arabidopsis thaliana* root exudates.**

Average on numbers of viable cells (Log_10_CFU/mL) for single cultures and co-cultures of *Paraburkholderia phytofirmans* PsJN, *Cupriavidus pinatubonensis* JMP134, *C. metallidurans* CH34 and *C. taiwanensis* LMG19424) grown on *A. thaliana* root exudates (PRE) collected at day 14, or 21, with plants exposed to standard or N-limiting conditions (14d.PRE, 21d.PRE and 21d.N-PRE). Each value represents averages from three replicates. Standard deviations were lower than 5 or 10% and are not shown for clarity.

| ***Burkholderiaceae* strains** | **PsJN** | | | **JMP134** | | | **CH34** | | | **LMG19424** | | |
| --- | --- | --- | --- | --- | --- | --- | --- | --- | --- | --- | --- | --- |
| *A. thaliana* exudates | 14d PRE | 21d PRE | 21d N-PRE | 14d PRE | 21d PRE | 21d N-PRE | 14d PRE | 21d PRE | 21d N-PRE | 14d PRE | 21d PRE | 21d N-PRE |
|  |  |  |  |  |  |  |  |  |  |  |  |  |
| Single (Log_10_CFU/mL) | 7.6 | 8.5 | 11.3 | 7.9 | 10.4 | 11.7 | 8.6 | 9.2 | 11.7 | 7.6 | 8.5 | 11.3 |
| Pairs |  |  |  |  |  |  |  |  |  |  |  |  |
| PsJN-JMP134 | 8.6 | 8.9 | 10.9 | 8.9 | 8.6 | 11.0 |  |  |  |  |  |  |
| PsJN-CH34 | 9.1 | 9.1 | 11.5 |  |  |  | 8.6 | 8.6 | 11.0 |  |  |  |
| PsJN-LMG19424 | 8.6 | 8.1 | 11.5 |  |  |  |  |  |  | 8.6 | 8.6 | 5.9 |
| JMP134-CH34 |  |  |  | 8.6 | 8.6 | 11.3 | 7.6 | 7.6 | 11.1 |  |  |  |
| JMP134-LMG19424 |  |  |  | 8.6 | 8.6 | 11.2 |  |  |  | 7.6 | 7.5 | 5.5 |
| CH34-LMG19424 |  |  |  |  |  |  | 8.2 | 7.9 | 11.2 | 8.1 | 7.9 | 11.0 |
| Trios |  |  |  |  |  |  |  |  |  |  |  |  |
| PsJN-JMP134-CH34 | 9.1 | 8.9 | 11.5 | 8.9 | 8.5 | 11.3 | 8.6 | 8.6 | 11.0 |  |  |  |
| PsJN-JMP134-LMG19424 | 8.4 | 8.2 | 9.8 | 8.6 | 7.9 | 10.5 |  |  |  | 8.2 | 8.1 | 5.2 |
| PsJN-CH34-LMG19424 | 9.6 | 9.1 | 10.7 |  |  |  | 8.5 | 8.6 | 11.5 | 8.6 | 8.7 | 7.4 |
| JMP134-CH34-LMG19424 |  |  |  | 8.6 | 8.6 | 11.4 | 7.6 | 7.9 | 5.9 | 7.9 | 7.6 | 8.3 |
| Quartet |  |  |  |  |  |  |  |  |  |  |  |  |
| PsJN-JMP134-CH34-LMG19424 | 8.1 | 8.9 | 11.1 | 7.9 | 8.5 | 9.8 | 7.3 | 7.6 | 10.8 | 8.1 | 7.9 | 7.5 |
