# Supplementary material for: Individual competence predominates over host nutritional status in Arabidopsis root exudate-mediated bacterial enrichment in a combination of four Burkholderiaceae species
Source: BMC Microbiol. 2022 Sep 17;22:218. doi: 10.1186/s12866-022-02633-8 (PMC9482264; doi:10.1186/s12866-022-02633-8)
Supplement: Supplementary file 2 — Additional file 2. Selection of replicates of the 4-member combination to set the next generation inocula. [file 12866_2022_2633_MOESM2_ESM.docx]

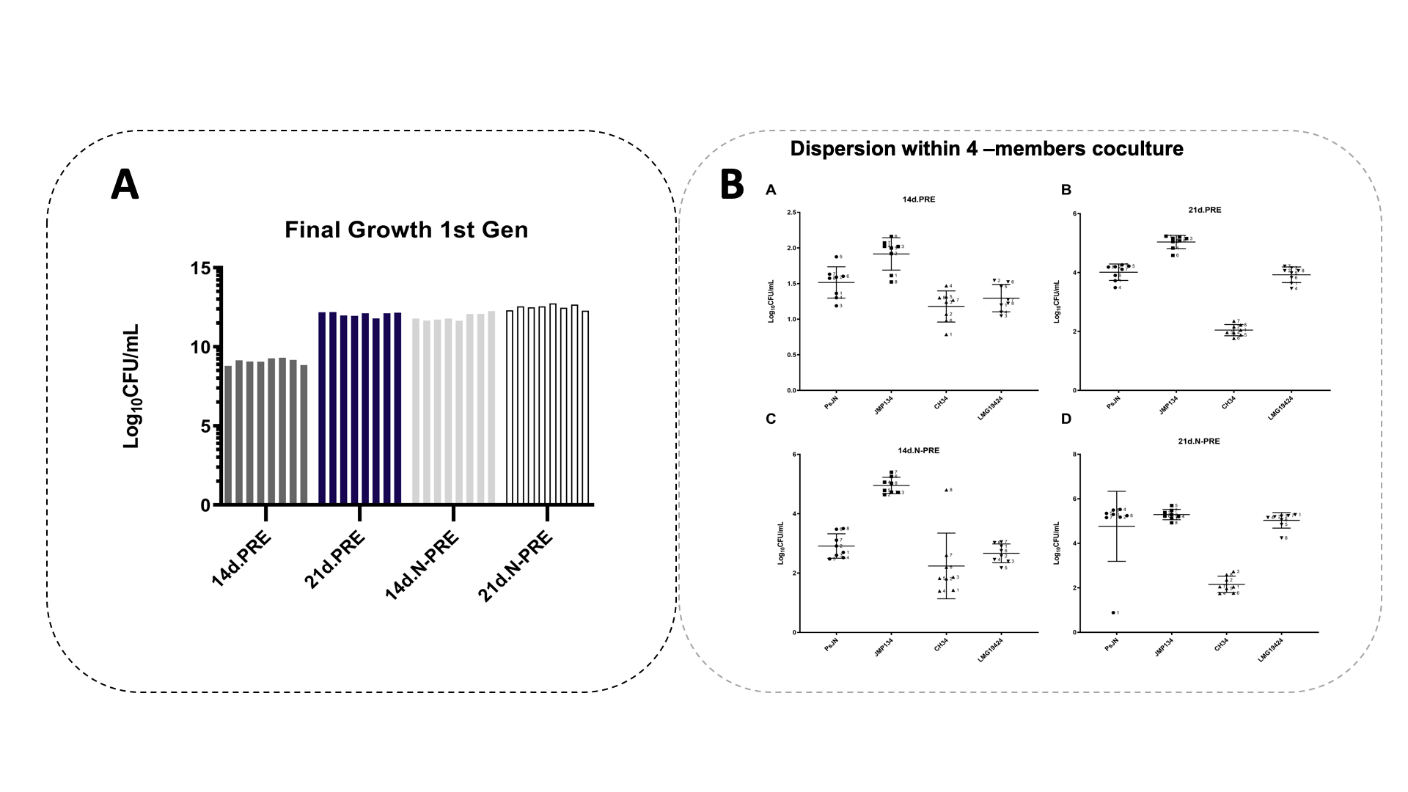


**Additional File 2. Selection of replicates of the 4-member combination to set the next generation inocula.** Final growth levels of individual members of the combination (*Paraburkholderia phytofirmans* PsJN, *Cupriavidus pinatubonensis* JMP134, *C. metallidurans* CH34, *C. taiwanensis* LMG19424) on each *Arabidopsis thaliana* root exudate, collected at day 14 or 21, under standard or N-limiting conditions (14d.PRE, 21d.PRE, 14d.N-PRE, and 21d.N-PRE), were determined by viable cell counting [colony forming units (CFU)/mL]. These growth levels were compared, and the three more abundant selected (**A**). Then, the dispersions of the growth values for each bacterium with respect to the growth of the total 4-member combination were evaluated **(B)**. Each dot represents a different replicate and those three with lower dispersion were selected.
