# Supplementary material for: Individual competence predominates over host nutritional status in Arabidopsis root exudate-mediated bacterial enrichment in a combination of four Burkholderiaceae species
Source: BMC Microbiol. 2022 Sep 17;22:218. doi: 10.1186/s12866-022-02633-8 (PMC9482264; doi:10.1186/s12866-022-02633-8)
Supplement: Supplementary file 3 — Additional file 3 Growth curves for individual and 4-member coculture of Burkholderiaceae strains. [file 12866_2022_2633_MOESM3_ESM.docx]

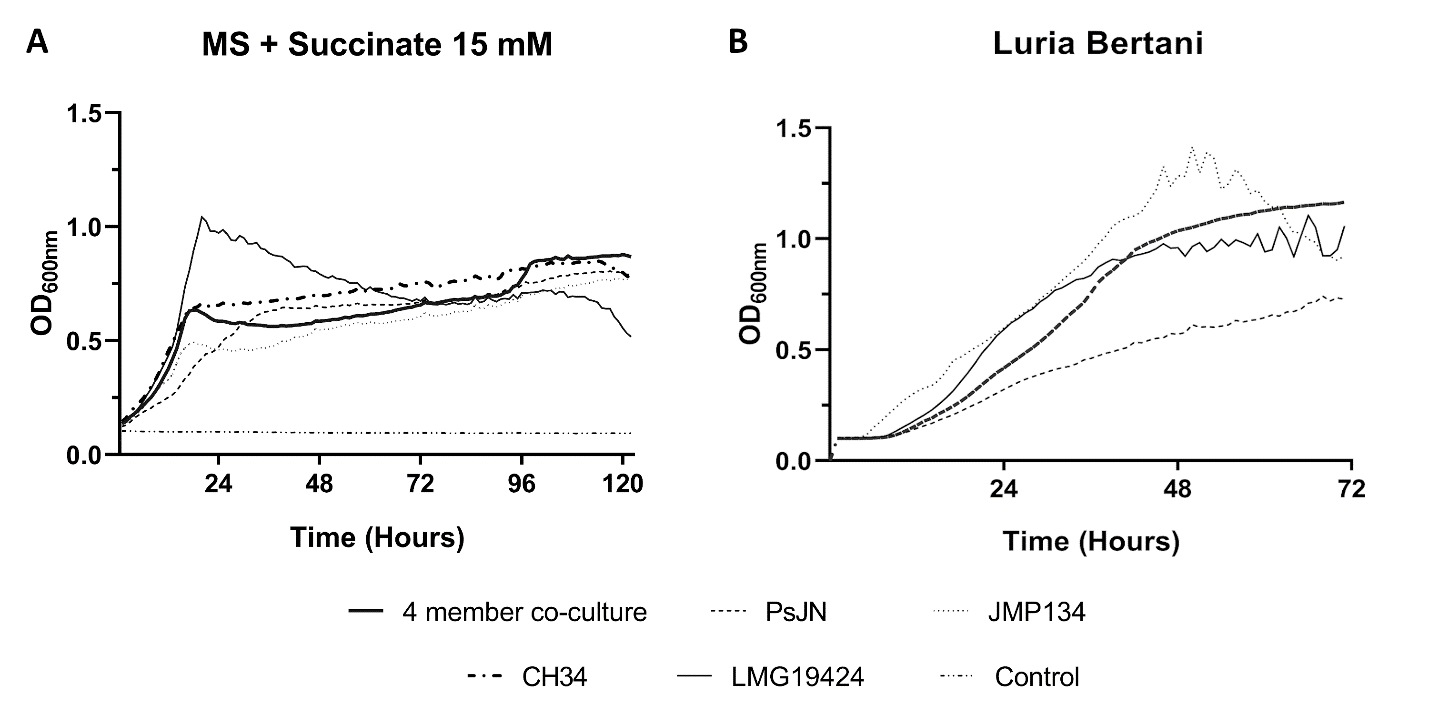


**Additional File 3***.* **Growth curves for individual and 4-member coculture of *Burkholderiaceae* strains.**

Growth curves obtained after 120 hours in culture tests carried out on 100% Murashige & Skoog with or without the addition of 15mM of succinate (A), or 72 hours on Luria – Bertani medium (B). Each growth curve corresponds to the average of three replicates of cultures inoculated with each individual strain alone, or the 4-member combination of *Paraburkholderia phytofirmans* PsJN, *Cupriavidus pinatubonensis* JMP134, *C. metallidurans* CH34, and *C. taiwanensis* LMG19424. Standard deviations were lower than 5 - 10% and are not shown for clarity.
