# Supplementary material for: Individual competence predominates over host nutritional status in Arabidopsis root exudate-mediated bacterial enrichment in a combination of four Burkholderiaceae species
Source: BMC Microbiol. 2022 Sep 17;22:218. doi: 10.1186/s12866-022-02633-8 (PMC9482264; doi:10.1186/s12866-022-02633-8)
Supplement: Supplementary file 4 — Additional file 4. Graphic representation of the elbow method to discriminate number of clusters. [file 12866_2022_2633_MOESM4_ESM.docx]

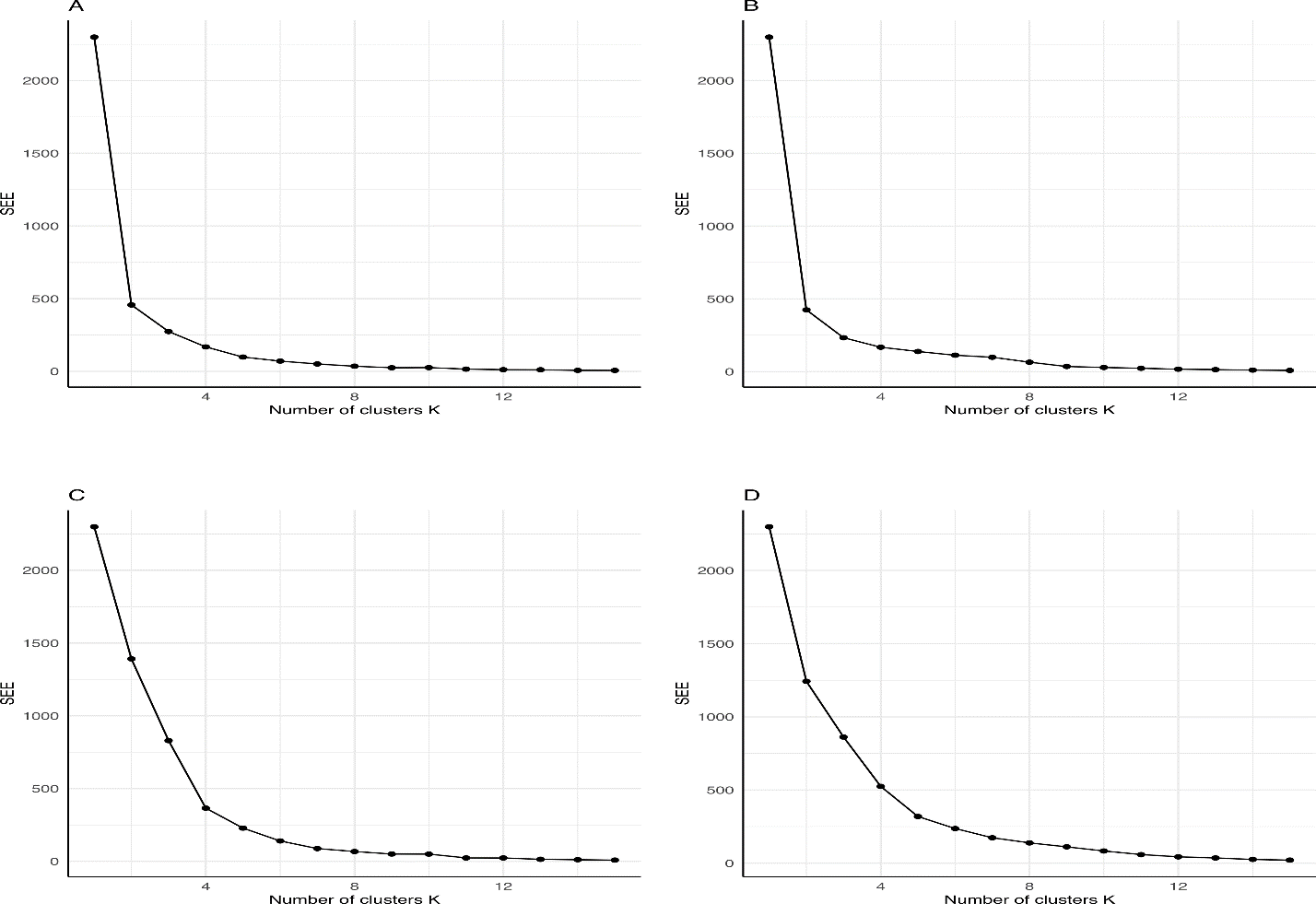


**Additional File 4***.* **Graphic representation of the elbow method to discriminate number of clusters***.* Panels (A) 14d.PRE, (B) 21d.PRE, (C) 14d.N-PRE, and (D) 21d.N-PRE shows the elbow method to discriminate the number of clusters used. On each panel, 20 growth curves were analyzed to obtain the clusters: eight replicates of the initial 4-member combination (Consortium_1 to Consortium_8) and three replicates for each individual culture: PsJN_1 to PsJN_3 for *Paraburkholderia phytofirmans* PsJN; JMP134_1 to JMP134_3 for *Cupriavidus pinatubonensis* JMP134; CH34_1 to CH34_3 for *C. metallidurans* CH34, and LMG19424_1 to LMG19424_3 for *C. taiwanensis* LMG19424.
